# Illogical Terminology

**Published:** 1904-10-15

**Authors:** E. T. Loeffler


### ILLOGICAL TERMINOLOGY.

BY E. T. LOEFFLER, B. S. D. D. S.

Read before the Jackson, Michigan, City Dental Society.

The logical application of the meaning of technical terms is, perhaps, one of the most interesting as well as the most important features of dental as well as medical science. There is, apparently, a lack of uniformity in the meaning and application of scientific terms. This is true, to a certain extent, at least, in every science; but since the advent of modern aseptic surgery, there has arisen an amount of discrepancy in regard to the signification and use of certain terms that is somewhat amazing in view of the general intelligence on this important subject.

After investigating this subject carefully, I am in doubt whether we shall be able to find a better and clearer verification of the above statements than could be found in the application of the terms, Antiseptic, Disinfectant, Germicide and Sterilize.

In order to discuss this subject intelligently and systematically, we will first of all give a definition of each of these terms by one of our most reliable authorities. According to Professor Novy of the University of Michigan, "An antiseptic is a drug, chemical substance, or agent, that will retard or inhibit the growth or development of micro-organisms." A disinfectant is a drug, chemical substance, or agent that will destroy or devitalize all pathogenic germs." A germicide is a drug, chemical substance, or agent that will destroy all active germs, "and to sterilize means to destroy all active germs as well as spores." I hope you will pardon any apparently unnecessary repetition; but, to make absolutely plain the distinction between an antiseptic and a disinfectant let me quote from Professor Novy's work on bacteriology, page 527, in which he states: "The first action of a chemical substance, when added to a recently inoculated culture medium, is to inhibit the growth of the organism. If the chemical substance is highly poisonous and is present in sufficient amount it will evidently kill the bacteria present. On the other hand, many weak substances, commonly designated as preservatives, will prevent the development of bacteria, but are not able to destroy them."

It is necessary, therefore, to clearly understand the distinction between an antiseptic and a germicide. The latter kills the growth, whereas the former prevents its further development. A germicide, when sufficiently diluted, may act as an antiseptic.

Again Professor Abbott, in his "Principles of Bacteriology," makes very plain the difference in meaning between the terms sterilization and disinfection. As he says: "In many laboratories it is customary to employ the term sterilization for the destruction of bacteria by heat, and the term disinfection for the accomplishment of the same end through chemical agents. This distinction in the use of the terms is not strictly correct, as we shall endeavor to explain.

The laboratory application of the term sterilization for the destruction of bacteria by high temperature probably arose from the circumstances that culture media, and certain other articles that it is desirable to render absolutely free from bacteria life, are not treated by chemical agents for this purpose, but are exposed to the influence of heat in various forms of apparatus known as sterilizers: and the process is therefore known as sterilization. On the other hand, cultures no longer useful, bits of infected tissue and apparatus generally, that it is desirable to render free from danger, are commonly subjected for a time to the action of chemical compounds possessing germicidal properties, that is, to the action of disinfectants; and the process is, therefore, known as disinfection, though the same end can also be attained by the application of heat to these articles.

Strictly speaking, sterilization implies the complete destructiou of the vitality of all micro-organisms that may be present in and about or upon the substance to be sterilized, and can be accomplished by the proper application of either thermal or chemical agents. Disinfection, may not necessarily involve the destruction of all living forms that are present, but only those possessing the power of infection. It may or may not, therefore, be complete in the sense of sterilization.

From this we see it is possible to accomplish both sterilization and disinfection by chemical or by thermal means."

Abbott states, page 70, of the above mentioned text: "An antiseptic is a body which, by its presence, prevents the growth of bacteria without of necessity killing them. A body may be an antiseptic without possessing disinfectant properties to any high degree, but a disinfectant is always an antiseptic as well. This is true, however, only to a limited extent."

Crookshank, in his text-book on bacteriology, states the following: "Agents which retard the growth of bacteria are generally spoken of as antiseptic, as distinguished from disinfectants which destroy their vitality. Though chemical disinfectants, germicides, well diluted, act as efficient antiseptics, the converse, that an antiseptic in sufficiently concentrated form will act as a disinfectant, is not the case.

The term, antiseptic, indeed, should be restricted to those substances or agents, which arrest the changes bacteria produce, but which do not prevent their springing into activity when removed to favorable conditions.

Thus excessive heat, which destroys bacteria and their spores, is a true disinfectant; and excessive cold, which only benumbs them, retarding their developement without killing them, is an antiseptic.

Perhaps I shall tire you, but I cannot help quoting from our illustrious bacteriologist, Sternberg, who makes the following paragraph very emphatic:

"The term antiseptic is used by some authors to designate an agent which destroys the vitality of the micro-organisms which produce septic decomposition and others of the same class. We prefer to restrict the use of the term to those agents which restrain the development of such micro-organisms without destroying their vitality. The complete destruction of vitality is effected by germicides or disinfectants."

This certainly goes to show that wide discrepancies prevail in the use of these technical terms.

To be a little more specific, I beg to call your attention to a few-paragraphs in Dr. Miller's work on "Micro-organisms of the Human Mouth," beginning at the bottom of page 227, which reads as follows:

"In connection with the antiseptic materials used in the human mouth, the question of their adaptability demands particular consideration; and in regard to this point I have examined a number of materials used in the treatment of the human mouth in the same form and concentration as may be made use of in the form of a mouth-wash. Usually in rinsing the mouth the solution remains from a few seconds to at most a minute in connection with the mucous membrane and the teeth; and we accordingly, for the purpose of sterilizing the oral cavity, use a material which in the adopted concentration is able to devitalize bacteria inside of a minute." I wish to call your attention to the use of the term antiseptic in this paragraph. An antiseptic, as we have endeavored to show, is not intended to devitalize germs but only to retard their growth, and according to leading authorities it ought to possess staying qualities, have a pleasant odor and taste, be non-irritating and not injurious to the the surrounding tissues. These are qualities that are hard to combine in any single agent. As Dr. Miller says: "The preparation of a mouth-wash which possesses an antiseptic action of any importance is accompanied with the greatest difficulties."

The term disinfectant, or perhaps germicide, ought to have been used in this connection, because evidently Dr. Miller had in mind a solution that would destroy or devitalize. However, the use of so strong an agent in a mouth-wash is impracticable. Again he uses the term sterilize, when in fact there is absolutely no necessity for such a potent remedy. To sterilize means to kill all germs including spores and that would mean death to the host, perhaps, as well as to the guest.

To show you that this term is really not to be used, let me refer you to page 232 of the same text-book mentioned in which the Doctor says "A solution which devitalizes spores in one minute is out of the question, and in fact is not at all necessary, since the conditions which lead to the formation of spores do not exist in the mouth where we find almost exclusively the vegetative forms."

In case of Dr. Miller this may have simply been an oversight. However, we find similar mistakes in other text-books in which this subject is brought up. For example, Grant in his new treatise: "Surgical Diseases of the Face, Mouth, and Jaws," page 14, states the following: "By antiseptics is meant the destruction of colonies of bacteria, etc." The term disinfection means destruction of germs, and ought to have been used in place of antiseptic.

On page 15 of the same text we have the following paragraph: "As antiseptics or germ-destroyers on living tissue, such solutions as have been mentioned, while not destroying all the germs in the tissues render the soil less favorable for their nutrition and thus prevent and retard colonization."

Here we can plainly see that an effort is made to increase the elasticity of the term antiseptic so as to include the power of a disinfectant.

A chemical substance like carbolic acid, for example, may be used either as an antiseptic or a disinfectant by simply varying the strength.

On the other hand permanganate of potash and hydrogen peroxide, which are excellent disinfectants and germicides, are absolutely worthless as antiseptics on account of their transitory character.

As I have stated before, all disinfectants may, to a certain extent at least, act as antiseptics; but no antiseptic can in any sense be considered a disinfectant or a germicide.

Take, if you please, a concentrated solution of sugar or salt will prevent putrefactive decomposition in organic material; but these do not destroy the vitality of the germs.

As another illustration, bichloride of mercury, 1.300000, will prevent the development of anthrax spores, but to destroy their vitality a solution as strong as 1.1000, must be used. As a rule, however, the difference existing between the restraining action of an antiseptic and the power of a germicide is not so great as above stated.

What are some of the essential qualities of a good and useful antiseptic? 1st, it must have staying quality or be only slightly soluble, otherwise it will be absorbed too readily and its local action is nil; 2nd, non-irritating, because it must remain in contact with the tissues for a long time; 3rd, only slightly toxic, so that it may not produce constitutional symptoms, in case of absorption; 4th, should not discolor the teeth; and 5th, it should have a pleasant odor and taste.

These are qualities hard to secure in any single solution. For example, bichloride of mercury, one of the best antiseptic solutions we possess, is an irritant; it discolors the teeth and has a most disagreeable metallic taste and should not be used except, perhaps, in cases of oral syphilis. Formaldehyde as a forty-percent solution, is a useful preparation, but as an antiseptic it is altogether too irritating.

And so we might go over the whole list of useful drugs and perhaps fail to find any that would have all the requisite qualities.

Miquel gives the following table of some of the best antiseptics:

| | | | | |
|---|---|---|---|---|
| Mercuric Iodide, efficient in the proportion | | | | 1.49000 |
| Silver Iodide | " | " | " | 1.33000 |
| Hydrogen Peroxid | " | " | " | 1.20000 |
| Mercuric Chlorid | " | " | " | 1.14300 |
| Silver Nitrate | " | " | " | 1.12500 |

Bernard Bennette, of Edinburgh, in an excellent article entitled, "A Useful Antiseptic," conveys a wrong impression of his idea of an antiseptic by the following closing statement: "I regard it as next in value as an all-round antiseptic and germicide to hydrogen peroxide."

In conclusion, I might add that, as professional men, we ought to be more accurate in the use of terms. This is especially applicable in the study of dental therapeutics in which more stress ought to be laid upon the proper use of the proper remedy.

